# Pre-existing immunity to SARS-CoV-2 before the COVID-19 pandemic era in Cameroon: A comparative analysis according to HIV-status

**DOI:** 10.3389/fimmu.2023.1155855

**Published:** 2023-03-08

**Authors:** Abba Aissatou, Joseph Fokam, Ezechiel Ngoufack Jagni Semengue, Désiré Takou, Aude Christelle Ka’e, Collins Chenwi Ambe, Alex Durand Nka, Sandrine Claire Djupsa, Grâce Beloumou, Laura Ciaffi, Michel Carlos Tommo Tchouaket, Audrey Rachel Mundo Nayang, Willy Leroi Togna Pabo, René Ghislain Essomba, Edie G. E. Halle, Marie-Claire Okomo, Anne-Cecile ZK. Bissek, Rose Leke, Yap Boum, Georges Alain Etoundi Mballa, Carla Montesano, Carlo-Federico Perno, Vittorio Colizzi, Alexis Ndjolo

**Affiliations:** ^1^ Laboratory of virology, Chantal BIYA International Reference Center for Research HIV/AIDS Prevention and Management, Yaoundé, Cameroon; ^2^ Serology Unit, Garoua Regional Health Centre, Garoua, Cameroon; ^3^ Faculty of Health Sciences, University of Buea, Buea, Cameroon; ^4^ Laboratory Unit, Operations sections, National Public Health Emergency Operations Coordination Centre, Yaounde, Cameroon; ^5^ Faculty of Medicine and Biomedical Sciences, University of Yaounde I, Yaounde, Cameroon; ^6^ Department of Biology, University of Rome “Tor Vergata”, Rome, Italy; ^7^ Department of Science and Technology, Evangelical University of Cameroon, Bandjoun, Cameroon; ^8^ Department of General Medicine, Mvangan District Hospital, Mvangan, Cameroon; ^9^ Project Coordinator, National Agency for Research on AIDS and Viral Hepatitis, Yaounde, Cameroon; ^10^ School of Health Sciences, Catholic University of Central Africa, Yaounde, Cameroon; ^11^ National Public Health Laboratory, Ministry of Public Health, Yaounde, Cameroon; ^12^ Division of Health Operational Research, Ministry of Public Health, Yaounde, Cameroon; ^13^ The Biotechnology Center of the University of Yaounde I and the Ministry of Scientific Research, Yaounde, Cameroon; ^14^ Division of Disease, Epidemic and Pandemic Control, Ministry of Public Health, Yaounde, Cameroon; ^15^ Department of Microbilogy, Bambino Gesu Pediatric Hospital, Rome, Italy

**Keywords:** HIV, SARS-CoV-2, immunoglobulin G/M, T-CD4 lymphocytes, HIV viral load

## Abstract

**Background:**

The lower burden of COVID-19 in tropical settings may be due to preexisting cross-immunity, which might vary according to geographical locations and potential exposure to other pathogens. We sought to assess the overall prevalence of SARS-CoV-2 antibodies and determine SARS-CoV-2 seropositivity according to HIV-status before the COVID-19 pandemic era.

**Methods:**

A cross-sectional and comparative study was conducted at the Chantal BIYA International Reference Centre (CIRCB) on 288 stored plasma samples (163 HIV-positive versus 125 HIV-negative); all collected in 2017-2018, before the COVID-19 pandemic era. Abbott Panbio™ COVID-19 IgG/IgM assay was used for detecting SARS-CoV-2 immunoglobulin G (IgG) and M (IgM). Among people living with HIV (PLHIV), HIV-1 viral load and TCD4 cell count (LTCD4) were measured using Abbott Real Time PCR and BD FACSCalibur respectively. Statistical analyses were performed, with p<0.05 considered statistically significant.

**Results:**

The median [IQR] age was 25 [15-38] years. Overall seropositivity to SARS-CoV-2 antibodies was 13.5% (39/288) of which 7.3% (21) was IgG, 7.3% (21) IgM and 1.0% (3) IgG/IgM. According to HIV-status in the study population, SARS-CoV-2 seropositivity was 11.0% (18/163) among HIV-positive versus 16.8% (21/125) among HIV-negative respectively, p=0.21. Specifically, IgG was 6.1% (10/163) versus 8.8% (11/125), p=0.26; IgM was 5.5% (9/163) versus 9.6%, (12/125), p=0.13 and IgG/IgM was 0.6% (1/163) versus 1.6% (2/125) respectively. Among PLHIV, SARS-CoV-2 seropositivity according to CD4 count was 9.2% (≥500 cells/µL) versus 1.8% (200-499 cells/µL), (OR=3.5; p=0.04) and 0.6% (<200 cells/µL), (OR=17.7; p<0.01). According to viral load, SARS-CoV-2 seropositivity was 6.7% (≥40 copies/mL) versus 4.9% (<40 copies/mL), (OR= 3.8; p<0.01).

**Conclusion:**

Before COVID-19 in Cameroon, cross-reactive antibodies to SARS-CoV-2 were in circulation, indicating COVID-19 preexisting immunity. This preexisting immunity may contribute in attenuating disease severity in tropical settings like Cameroon. Of relevance, COVID-19 preexisting immunity is lower with HIV-infection, specifically with viral replication and poor CD4-cell count. As poor CD4-count leads to lower cross-reactive antibodies (regardless of viral load), people living with HIV appear more vulnerable to COVID-19 and should be prioritized for vaccination.

## Introduction

December 2019 was marked by the appearance of the new coronavirus 2019 disease (COVID-19) in the city of Wuhan in China, which quickly became a pandemic resulting on January 30^th^ 2020 in a Public Health Emergency of International Concern (PHEIC) (1–4). As of November 22^nd^ 2022, 643,620,075 cases had been diagnosed worldwide with 6,628,090 deaths reported, giving a fatality rate of 1.03% three years after the beginning of the pandemic (5). Seven coronaviruses can lead to infection, but the causal agent of COVID-19 was identified as the novel severe acute respiratory syndrome coronavirus-2 (SARS-CoV-2) (3, 6, 7). Of note, SARS-CoV-2 is an enveloped virus with a linear and unsegmented positive-sense RNA genome, coding for four main structural proteins, namely: the spike (S), nucleocapsid (N), envelope (E) and membrane (M) (8). The S protein contains, the S1 subunit which is divided into an N-terminal domain (NTD) and a receptor binding domain (RBD) responsible for binding the virus to the host cell Angiotensin-Converting Enzyme 2 (ACE2) receptor binding domain (8). The N protein on the other hand is involved in the externalization of viral particles from the infected cell. Additionally, the spike glycoprotein polymer (specifically S1) mediates viral attachment, followed by membrane fusion. This glycoprotein is immunogenic and hence ideal for serosurveys (targeting IgM and IgG humoral circulating antibodies) (3, 7, 9–11), which are one of the best approaches to appraise the extent of COVID-19 infection and disease circulation within a given community.

Surprisingly, from the overall global burden of COVID-19, a small proportion of cases were reported in Africa. On November 22^nd^ 2022, 12,697,321 cases were reported indicating 1.97% of the global burden (5) and suggesting a much lower severity of the disease (12). Several hypotheses have been proposed to explain this low incidence of COVID-19 in Africa among which were: (i) the young population in Sub-Saharan Africa as compared to Caucasians; (ii) SARS-CoV-2 persistence and spread disadvantaged by climatic and environmental factors; (iii) social distancing favored by the lifestyle in rural/less developed areas, which limits the spread of the disease; (iv) underestimation of morbidity and mortality counts due to poor testing coverage, reflecting weak health systems; (v) a rapid activation of the natural innate non-specific immunity due to an overexposure to pathogens; and (vi) specific immune response following a previous contact with viruses sharing common antigenic profiles with SARS-CoV-2 (4, 6, 8). The potential low circulation of SARS-CoV-2 in Africa may be justified by the existence of specific pre-pandemic antibodies, responsible for cross-immunity during the pandemic (8). Furthermore, with the emergence in 2019 of SARS-CoV-2, the world now has to face two pandemics: COVID-19 and HIV/AIDS (13). As COVID-19 pandemic continues to cause much uncertainty around the world, and especially among people living with and affected by HIV (>67% of whom residing in sub-Saharan Africa), understanding its extent as well as the determinants of induced immunogenicity is crucial to frame the response strategy within national programs and better prepare for future pandemics. We therefore sought to assess the overall prevalence of SARS-CoV-2 antibodies and determine SARS-CoV-2 seropositivity according to HIV-status during the COVID-19 pre-pandemic era in Cameroon.

## Methods

### Study design

We carried out a case-control study on archived plasma samples collected between 2017 and 2018, before the outbreak of COVID-19 in Cameroon.

### Study site

As a COVID-19 reference center for diagnostic and genomic surveillance, the “Chantal BIYA” International Reference Centre for research on HIV/AIDS prevention and management (CIRCB) is a government institution of the Cameroonian Ministry of Public Health committed to research on HIV/AIDS prevention and management. Additionally, CIRCB covers: (a) HIV early infant diagnosis in the frame of the national PMTCT program; (b) diagnosis of co-infections with HIV; (c) viral load measurement; (d) CD4 and CD8 T lymphocytes counts; (e) biochemical and hematological tests for drug follow-up; (f) genotypic HIVDR testing (GRT) at subsidized costs; with quality control programs conducted in partnership with Quality Assessment and Standardization of Indicators (QASI) and other international organizations (http://www.circb.cm/btc_circb/web/).

### Sampling and eligibility criteria

We enrolled archived plasma samples previously anonymized and codified as per institutional biobanking procedures (from both HIV-positive and negative individuals). Socio-demographic and clinical data were obtained from the CIRCB database. Samples were from people residing in the Centre region of Cameroon. For HIV-positive individuals, only those with CD4-cell count, HIV-1 viral load measurements and with a complete treatment history were included; those with any co-infection or related co-morbidity were excluded.

### Clinical and laboratory procedures

#### Serological assays

SARS-CoV-2 antibodies were tested using the Abbott Panbio™ COVID-19 IgG/IgM Rapid Test Device as per manufacturer’s instructions. Assay performance as reported by the manufacturer was as follows: sensitivity (97.8%); specificity (92.8%); precision (>99% both for intra-assay and inter-assay assessments) (14). Briefly, plasma samples were mixed by low-speed vortex after which 10μL of supernatant was applied to the specimen well (S) of the test device. Two drops (approximately 60 μL) of buffer were added and a timer was started for 10 minutes. At the end of the 10 minutes the test device was read. A valid result consisted of the appearance of a red line in the Control (C) area of the reading window. A negative result consisted of the presence of the red line only in the C area whereas the presence of a red line on both C and G (IgG) areas indicated a reactivity to IgG; a red line on both C and M (IgM) areas, reactivity to IgM and a red line in C and both G and M areas of the reading window indicated a positive result for both IgG and IgM.

#### T-CD4 lymphocytes phenotyping

Before storage in the biobank, CD4 cells count of PLHIV had also been performed using the BD FACSCalibur™ Flow Cytometer as per the manufacturer’s instructions with results reported as the number of cells per microliter of blood (15). We therefore classified CD4 results as follows: no immunodeficiency (above or equal to 500 cell/µL); mild immunodeficiency (between 350 and 499 cell/µL); advanced immunodeficiency (between 200 and 349 cell/µL) and severe immunodeficiency (below 200 cell/µL).

#### Viral load measurements

Before storage in the biobank, HIV-1 viral load measurement had also been performed using the Abbott™ *m2000rt* instrument for Real Time PCR as-per the manufacturer’s instructions (16), with a lower detection threshold of 40 HIV-1 RNA copies/mL and an upper detection threshold of 10,000,000 copies/mL.

#### Statistical analysis

Data collected were entered on Microsoft Excel 2021. The software IBM.SPSS^®^ Statistics V.20 was used for statistical analysis. Association analyses were performed using Pearson Chi-Square Test with statistical significance considered at p<0.05. All variables with p<0.2 during bivariate analyses were retained for multivariable analysis.

## Results

### Clinical characteristics of the study population

Out of a total of 288 selected samples, 163 were HIV positive (56.6%) and 125 HIV negative (43.4%). Median age [interquartile range; IQR] in the study population was 25 [15-38] years; 18 [13-41] years among HIV-positive versus 28 [23-35] years among HIV negative. Majority of our study population was of the male gender, 58.0% (159/288), with similar distribution according to HIV-status. The rest of socio-demographic and clinical parameters are summarized in **Table 1**.

**Table 1:** Clinical characteristics of the study population.

Variables		Effective n (%)
*Gender*		
	Female	121 (42.0)
	Male	167 (58.0)
*Age*		
	Median age	25 years
	0-19	108 (37.5)
	20-39	118 (40.9)
	40-59	54 (18.8)
	>60	8 (2,8)
*HIV status*		
	HIV positive	163 (56.6)
	HIV negative	125 (43.4)
WHO clinical stages (for HIV positive) *		
	I	114 (75.5)
	II	21 (13.9)
	III	14 (9.3)
	IV	2 (1.3)
	Median ART-duration (months)	24 [7-66]
*ART line*		
	First line	139 (85.3)
	Second line	24 (14.7)
*Most prescribed regimen*		
	TDF+3TC+EFV (in first line)	56 (40.3)
	TDF+3TC+ATV/r (in second line)	9 (37.5)
*T-CD4 counts (cells/µL)*		
	≥ 500	65 (39.9)
	[350-499]	12 (7.4)
	[200-349]	26 (15.9)
	< 200	60 (36.8)
*HIV Viral Load (copies/mL)*		
	< 40	50 (30.7)
	≥ 40	113 (69.3)

*Data regarding WHO clinical stages were obtained for 151 participants only.

### Overall seroprevalence of SARS-CoV-2 antibodies

The global prevalence of SARS-CoV-2 antibodies was 13.5% (39/288) in the study population; 7.3% (21/288) IgG, 7.3% (21/288) IgM and 1.04% (3/288) IgG/IgM. Distribution of SARS-CoV-2 antibodies was similar according to age and HIV-status (p=0.07 and p=0.21 respectively); but statistically higher in men (aOR=2.54; [95%CI: 1.15-5.64]; p=0.02), as presented in **Tables 2**, **3** below.

**Table 2:** Overall prevalence of SARS-CoV-2 antibodies (IgG and IgM) using the rapid diagnostic assay.

Variables	Overall prevalence of Ig		Prevalence of IgG		Prevalence of IgM	
	n (%)	p-value	n (%)	p-value	n (%)	p-value
*Gender*						
* Male*	29 (17.37)	0.04	17 (10.18)	0.03	14 (8.38)	0.54
* Female*	10 (8.26)		4 (3.31)		7 (5.79)	
*Age*						
* <25 years*	25 (17.36)	0.08	14 (9.72)	0.17	13 (9.03)	0.36
* >25years*	14 (9.72)		7 (4.86)		8 (5.56)	
*HIV-status*						
* HIV+*	18 (11.04)	0.21	10 (6.13)	0.53	9 (5.52)	0.27
* HIV-*	21 (16.80)		11 (8.80)		12 (9.60)	

**Table 3:** Determinants of a high SARS-CoV-seroprevalence (multivariable analysis).

Variables	Adjusted odds ratio	95% C.I.	p-value
***Age (>25/<25)***	0.51	[0.25-1.06]	0.07
***Gender (M/F)***	2.54	[1.15-5.64]	0.02

### Seroprevalence of SARS-CoV-2 antibodies among HIV-infected participants

Concerning HIV-infected participants, virological control (VL<40copies/mL), high CD4 cells count (>350cells/µL) and ART duration >23 months were all significantly associated to SARS CoV-2 seropositivity in bi-variables analyses (p=0.005; p=0.01; p<0.001 respectively) but not in multivariable analysis (p=0.14; p=0.19; p=0.96 respectively) as presented in **Table 4** below.

**Table 4:** Predictors of SARS-CoV-2 seroprevalence among HIV-infected participants.

Variables	Prevalence of Ig		
	n (%)	OR [95%CI] (p-value)	aOR [95%CI] (p-value)
*CD4 (cells/µL)*			
* ≥350*	14 (18.18)	4.55 [1.42-14.51] (0.01)	2.65 [0.62-11.26] (0.19)
* <350*	4 (4.65)		
*Viral load (copies/mL)*			
* <40*	10 (20.41)	3.39 [1.25-9.23] (0.026)	0.58 [0.16-2.12] (0.41)
* ≥40*	8 (7.02)		
*ART-duration (months)*			
* < 23*	0 (0.00)	/(<0.001)	/(0.96)
* >23*	18 (22.78)		
*Treatment line*			
*1^st^ line*	13 (9.35)	0.39 [0.12-1.22] (0.19)	2.87 [0.85-9.66] (0.08)
*2^nd^ line*	5 (20.83)		

## Discussion

To the best of our knowledge, no evidence clearly explains the low severity of COVID-19 in Africa, a continent that has the weakest health system and infrastructures. Indeed, the limited number of healthcare infrastructures, the poor access to specialized health services (present only in some urban areas), and the limited number of professionals trained in critical care have already been widely documented across the continent (3, 17–21); thus calling for further investigations to clarify on this low incidence of COVID-19 in Africa (12). The objective of this study was to assess the circulation of SARS-CoV-2 antibodies in a pre-pandemic era according to HIV-infection in Cameroon.

First and foremost, our findings effectively demonstrated the circulation of SARS-CoV-2 antibodies in Cameroon before the disease outbreak in 2019. This pre-existence of specific SARS-CoV-2 antibodies strongly supports the hypothesis of potential cross-immunity during the pandemic. The later could also be justified by a previous contact with other coronaviruses sharing common antigenic profiles (4, 8), which could be either HKU1, NL63, OC43 or 229E. Moreover, the circulation of these specific coronaviruses within Central Africa had never been described before the COVID-19 outbreak (22, 23).. Assessing the titer and immunogenicity of these specific pre-pandemic SARS-CoV-2 antibodies through broadly neutralizing assays will help confirm this hypothesis and further characterize this cross-immunity to understand its effect on SARS-CoV-2 variants and sub-lineages circulating nowadays (24). On one hand, we observed a higher sero-positivity SARS-CoV-2 antibodies among males as compared to females, likely driven by the differential level of ACE-2 receptors among men as compared to women (8, 25). On the other hand, knowing that estrogens decrease plasma renin activity while androgens increase plasma renin activity and the expression of angiotensinogen messenger RNA, it is therefore predicted that the latter hormone would up-regulate ACE2 expression (25). In effect, the human angiotensin-converting enzyme 2 (ACE2) has been described as the functional receptor for the severe acute respiratory syndrome caused by coronaviruses (8, 26), thus facilitating recognition and infection by coronaviruses (26). Therefore, the protective role of ACE2 in chronic pathologies like hypertension, cardiovascular diseases, and acute respiratory distress syndrome (25), would be reversed in the advent of COVID-19.

With respect to HIV-status, we observed here a similar distribution of SARS-CoV-2 antibodies between HIV-infected and uninfected participants. This observation is in line with several studies conducted early in the pandemic, indicating that the clinical presentation of COVID-19 is similar in people with and without HIV, particularly if they are on ART and have achieved HIV viral suppression (13, 27–29). However, among sera of HIV-infected participants, high CD4 counts, virological control (>40copies/mL) and ART duration, seemed individually associated to the presence of SARS-CoV-2 antibodies; suggesting that people with a highly compromised immune system, an uncontrolled viral replication and/or the absence of an effective ART-regimen stand a high risk of not controlling SARS-CoV-2 replication or developing severe symptoms of COVID-19 (13). This observation therefore suggests that a closer monitoring of HIV/SARS-CoV-2 co-infection would be beneficial in order to preserve the benefits of vaccine-induced immunogenicity in this key population. Importantly, the pathogenicity associated to each virus separately and subsequent impairments to the immune system have been described; and even though vaccination of HIV-infected is essential to uphold an already weak immune system, it is important to emphasize that our findings primarily suggest there is a greater risk of COVID-19 severity among the severely immune-compromised and in those with an uncontrolled viral replication.

## Conclusion

In summary, cross-reactive antibodies to SARS-CoV-2 were in circulation in Cameroon before COVID-19, suggesting preexisting immunity, which may have contributed in limiting the spread of COVID-19 and attenuating the severity of new variants within this tropical setting. This preexisting immunity appears similar among HIV-infected versus uninfected participants. As poor CD4-count leads to lower cross-reactive antibodies (regardless of viral load), people living with HIV (especially those with poor clinical status) appear more vulnerable to COVID-19 and should be prioritized for vaccination.

## Data availability statement

The raw data supporting the conclusions of this article will be made available by the authors, without undue reservation.

## Ethics statement

The studies involving human participants were reviewed and approved by National Ethics Committee for Research on Human Health, Cameroon. Written informed consent to participate in this study was provided by the participants’ legal guardian/next of kin.

## Author contributions

JF and DT, conceptualization. AA, EN, DT, CC, ADN, AK’E, SD, and GB, formal analysis and investigation. AA, JF, EN, DT, CC, ADN, AK’E, MT, AM, and WT, data curation and methodology. AA, EN, DT, and JF, writing the original draft preparation. DT, SD, GB, LC, RE, EH, M-CO, A-CB, RL, YB, GE, CM, C-FP, VC, AN, AM, and WT, resources, supervision, and funding acquisition. AA, JF, EN, DT, CC, ADN, C-FP, VC, and AN, writing-review and editing. All authors contributed to the article and approved the submitted version.
